# Investigating metabolic syndrome markers and body mass index changes in patients with acne vulgaris treated with isotretinoin: A prospective study

**DOI:** 10.1111/jocd.16533

**Published:** 2024-08-14

**Authors:** Afsaneh Sadeghzadeh Bazargan, Alireza Jafarzadeh, Fatemeh Danandeh, Sepideh Salehi

**Affiliations:** ^1^ Department of Dermatology, Rasool Akram Medical Complex Clinical Research Development Center (RCRDC), School of Medicine Iran University of Medical Sciences (IUMS) Tehran Iran; ^2^ Skin and Stem Cell Research Center Tehran University of Medical Sciences Tehran Iran; ^3^ Faculty of Medicine Iran university of Medical Sciences Tehran Iran; ^4^ Department of Dentistry Shahid Beheshti University of Medical Sciences Tehran Iran

**Keywords:** acne, BMI, body mass index, Isotretinoin, metabolic syndrome

## Abstract

**Background:**

Acne vulgaris is a common inflammatory skin disease that occurs during puberty, affecting approximately 85% of young adults and may persist into adulthood. The pathophysiology of acne is multifactorial, involving hormonal, inflammatory, and immune mechanisms. Isotretinoin is widely used for treating severe cystic acne or recurrent acne. This medication is considered a pharmacological option that significantly reduces sebum secretion, leading to a reduction in the size of sebaceous glands. It also induces a lack of differentiation in sebaceous cysts, resulting in a decrease in lipid accumulation.

**Method:**

This research is a prospective study. Patient contact details were obtained directly from those visiting the dermatology clinic, and they were monitored for a duration of 3 months. Essential data was gathered through patient examinations and inquiries at the clinic, including the prescription of tests prior to initiating isotretinoin treatment. Furthermore, follow‐up tests and examinations were performed within the initial and third months post‐treatment commencement.

**Results:**

Sixty‐two patients participated in the study, selected through non‐probability (convenience) sampling. The therapeutic dose taken by patients was 20 mg of isotretinoin daily (*n* = 49) or every other day (*n* = 13). Among the participants, six patients experienced a decrease of 3 units or more in HDL levels, while 16 patients saw an increase of 3 units or more in LDL levels, 3 months after beginning the treatment. Additionally, the triglyceride (TG) levels increased by 9 units or more in six individuals, and the blood sugar (BS) levels increased by 5 units or more in nine individuals, 3 months after treatment initiation. Moreover, one person's waist circumference increased by 1.5 cm 3 months after treatment began. The average weight of the individuals at the end of the treatment rose from 60.74 kg to 61.12 kg. However, this weight increase was not statistically significant. (*p* > 0.05).

**Conclusion:**

In general, the results of our study show that the use of oral isotretinoin as a treatment option for the management of acne vulgaris can be effective when administered at the correct dosage, offering a safe and low‐complication option.

**Abbreviation**
BMI: Body mass index

## 1 INTRODUCTION

Acne is a chronic inflammatory disease that occurs as a result of involvement of the pilosebaceous units of the skin and commonly affects the adolescent population,^1^ but it also has a prevalence in adulthood^2^ and affects up to 87% of adolescents and 54% of adults (with varying degrees of severity).^1^ Studies have also shown that acne predominantly affects the facial area more than the trunk.^3^, ^4^ The pathophysiology of acne is multifactorial and involves hormonal, inflammatory, and immune mechanisms.^5^, ^6^ Other studies have also suggested that family history and lifestyle might be risk factors.^7^, ^8^

There are four processes that play a central role in the formation of acne lesions: inflammatory mediators released into the skin, alteration in the process of keratinization leading to the formation of comedones, increased and altered sebum production due to androgens (or increased sensitivity in androgen receptors), and follicular colonization by Cutibacterium acnes.^9^ However, the sequence and interaction between these events are not fully understood.^9^ It has been reported that the differentiation pathways in sebaceous glands and adipocytes may be similar. Therefore, a better understanding of sebaceous gland differentiation and lipogenesis, as well as potential therapeutic approaches for sebaceous disorders, may contribute to our knowledge of how adipocytes differentiate.^10^, ^11^

Without treatment or inadequate treatment, severe acne vulgaris can lead to the development of detrimental psychological and physical effects. Acne treatment improves the quality of life of patients and can prevent the formation of acne scars on the face.^12^, ^13^ Isotretinoin is widely used for the treatment of severe cystic acne or recurrent acne. This medication is considered a pharmacological option that has the ability to significantly reduce sebum secretion and thus can lead to a reduction in the size of sebaceous glands. It also induces a lack of differentiation in sebaceous cysts, resulting in a decrease in lipid accumulation. Despite common misperceptions, there is weak evidence for increased incidence of depression, suicidality, or inflammatory bowel disease with isotretinoin use.^14^ However, the precise mechanism of action of this drug is still not fully understood.^2^, ^15^

Laboratory and clinical studies have shown that retinoids affect the secretion of leptin from adipocytes, and this effect is not due to the reduction in adipocyte tissue size.^16^ Leptin has pleiotropic effects on the physiology and pathophysiology of energy homeostasis, endocrine glands, and metabolism, and numerous studies have found a positive correlation between leptin levels and obesity and the development of metabolic syndrome.^17^, ^18^ Given the potential problems that acne can cause for patients and the possible effects of using the medication for acne treatment on lipid profiles and inflammatory markers, including leptin in patients' bodies, as well as the concerns and questions many patients have about the drug's effects on their weight, we aimed to investigate changes in BMI and markers of metabolic syndrome in patients with severe acne vulgaris undergoing isotretinoin treatment at the Dermatology Clinic both before starting treatment and 3 months after treatment.

## 2 MATERIALS AND METHODS

### 2.1 Patients

The research is a prospective study that collected data from patients without intervening in their current conditions. Patients receiving isotretinoin treatment for severe acne vulgaris in 2019 and 2020 were selected using convenience sampling. The rationale for choosing convenience sampling lies in its ease of implementation, convenient access, and speed in selecting participants, as opposed to more precise sampling methods like random sampling. Convenience sampling is often cost‐effective, time‐efficient, and logistically simpler to execute.

The inclusion criteria comprised a diagnosis of severe acne vulgaris (nodulocystic acne, acne resistant to standard treatment, acne with a risk of scarring), completion of a 3‐month treatment regimen, attendance at follow‐up visits, and willingness to participate. The sample size was derived from all eligible patients, totaling 62. Exit criteria included specific health conditions and patient dissatisfaction. Contact information was gathered, and patients were scheduled for follow‐up visits at the dermatology clinic. Data collected before treatment included measurements such as height, weight, waist circumference, and metabolic tests.

### 2.2 Assessment method

One month and 3 months after drug consumption, the patients' waist circumference and weight were measured at the specified times, and the test results were obtained. The data was then prepared for statistical analysis. Metabolic syndrome was defined based on the following criteria: a waist circumference greater than 102 cm in men and 88 cm in women, hypertriglyceridemia with triglyceride levels ≥150 mg per deciliter, low HDL cholesterol levels <40 mg per deciliter in men and 50 mg per deciliter in women, and high FBS (fasting blood sugar) levels ≥100 mg per deciliter, use of diabetes medications, or a history of Type 2 diabetes in medical records.

### 2.3 Data analysis

All patients' data and information remained confidential. After extraction, the data were and assigned a code for each patient's information. Qualitative data were reported as percentages and frequencies, and quantitative data were reported as mean ± standard deviation (SD). The chi‐squared test was used to examine the relationship between qualitative variables. The *t*‐test was used to compare two means, and ANOVA was used to compare more than two means. Non‐parametric equivalent tests were employed if the data distribution was non‐normal. A significance level of 0.05 was considered. All data were analyzed using SPSS software version 26.

### 2.4 Ethical considerations

The participants in this project adhered to all Helsinki ethical principles. This research was approved by the Research Council with the ethics code number IR.IUMS.FMD.REC.1401.245.

## 3 RESULTS

The study included 62 Iranian patients, all diagnosed with severe acne vulgaris, who were prescribed a therapeutic dose of 20 mg of isotretinoin daily (*n* = 49) or every other day (*n* = 13). Among the participants, there were 55 women and 7 men. The mean age of the patients was 21.27 (±3.89) years, with women averaging 21.85 (±3.98) years and men averaging 18.85 (±2.03) years. Out of the 62 patients studied, 4 patients (6.45%) met the criteria for metabolic syndrome before the study. The comorbidities, listed in order of prevalence, included androgenetic alopecia in 37 patients (59.68%), polycystic ovary syndrome in 14 patients (22.58%), hypertension in 4 patients (6.45%), and asthma in 1 patient (1.61%). The remaining patients did not report any comorbidities.

The mean initial LDL was 81.85 (±20.84), HDL was 54.17 (±7.30), triglycerides were 94.24 (±33.39), blood glucose was 87.79 (±5.88), and waist circumference was 77.33 (±7.39). At the third month after treatment initiation, the mean LDL was 82.48 (±21.75), HDL was 54.04 (±7.30), triglycerides were 96.90 (±33.22), blood glucose was 88.01 (±5.75), and waist circumference was 77.53 (±7.30).

During the treatment, some patients experienced changes in their lipid profiles. Six patients had a decrease in HDL levels by 3 units or more, while 16 patients had an increase in LDL levels by 3 units or more. Additionally, triglyceride levels increased in six individuals by 9 units or more, and blood sugar (BS) levels increased in nine individuals by 5 units or more. One individual had an increase in waist circumference by 1.5 cm after 3 months of treatment.

The average weight at the end of treatment slightly increased but was not statistically significant. There were no significant differences in BMI, laboratory factors (LDL, HDL, triglycerides, and BS), or waist circumference before and after treatment. The frequency distribution of metabolic syndrome remained stable throughout the study. The percentage changes in factors related to serum lipids, waist circumference, weight, and blood glucose did not differ significantly between individuals with and without metabolic syndrome. (Tables 1, 2, 3, 4, 5).

**TABLE 1:** Mean BMI of individuals before treatment, one month after treatment initiation, and at final follow‐up.

Variables	Before the treatment	One month after treatment	Final follow‐up	*p*‐value (initial and final)
	Mean (standard deviation)	Mean (standard deviation)	Mean (standard deviation)	
BMI	22.30 (2.57)	22.31 (2.59)	22.44 (2.56)	1.00

**TABLE 2:** Mean laboratory factors of individuals before treatment, 1 month after treatment initiation, and at final follow‐up.

Variables	Before the treatment	One month after treatment	Final follow‐up	*p*‐value (initial and final)
	Mean (standard deviation)	Mean (standard deviation)	Mean (standard deviation)	
LDL	81.85 (20.84)	81.01 (21.37)	82.48 (21.75)	1.00
HDL	54.17 (7.30)	53.79 (7.17)	54.17 (7.30)	0.737
TG	94.24 (33.39)	95.37 (33.12)	96.90 (33.22)	1.00
BS	87.79 (5.88)	87.08 (5.53)	88.01 (5.75)	0.961

**TABLE 3:** Mean waist circumference of individuals before treatment, 1 month after treatment initiation, and at final follow‐up.

Variables	Before the treatment	One month after treatment	Final follow‐up	*p*‐value (initial and final)
	Mean (standard deviation)	Mean (standard deviation)	Mean (standard deviation)	
Waist circumference	77.33 (7.39)	77.36 (7.33)	77.53 (7.30)	1.00

**TABLE 4:** Comparison of the frequency distribution of metabolic syndrome in patients.

Variable	Before the initiation of treatment number(frequency)	First month after treatment number(frequency)	After 3 months number(frequency)	*p*‐value
Metabolic syndrome‐	6 (9.6%)	6 (9.6%)	6 (9.6%)	‐

**TABLE 5:** Comparison of mean quarterly changes in study variables in patients with metabolic syndrome and others.

Variables	Patients with metabolic syndrome(frequency)	Patients without metabolic syndrome (frequency)	*p*‐value
LDL	+3.3%	+0.3%	0.42
HDL	_0.6%	_0.4%	1.00
TG	+2.22%	+2.94%	0.856
BS	+1.3%	+1.8%	0.948
Weight	+0.22%	+0.06%	1.00
BMI	+0.17%	+0.6%	1.00
Waist circumference	+0.08%	+0.06%	1.00

## 4 DISCUSSION

Acne vulgaris is a common condition during adolescence and the most prevalent inflammatory disease of the sebaceous glands. One of the treatment approaches for alleviating acne is the use of low‐dose oral isotretinoin. Due to the teratogenic nature of this medication and its negative impact on the liver, numerous studies have been conducted to evaluate the safety and efficacy of isotretinoin in the treatment of moderate to severe acne vulgaris.^19^, ^20^

In our study, the mean age of the patients was 21.27 (3.89) years. This finding is consistent with previous studies indicating a higher prevalence of acne vulgaris in the adolescent population and early adulthood.^9^, ^21^ The mean age of women was higher than that of men. Additionally, the number of female patients was approximately eight times higher than that of male patients. Previous epidemiological studies have also reported a higher number of women compared to men.^3^, ^22^ This finding can be attributed to multiple and multifactorial factors. The higher prevalence of acne vulgaris in women compared to men may be due to women's greater attention to skin health and their higher rate of seeking medical care. It can also be attributed to the use of cosmetics and exogenous hormonal sources such as certain foods. However, further research is needed to understand the extent of the impact of each of these factors.

During the treatment with isotretinoin, monitoring of serum lipid levels in individuals is of great importance. For example, a study has shown that individuals who develop hypertriglyceridemia during isotretinoin treatment for acne are at a higher risk of future increases in blood lipid levels and metabolic syndrome.^23^ In our study, despite a slight decrease in the mean of HDL (−0.18%) and an increase in LDL (0.76%), triglycerides (2.82%), and blood glucose (0.25%) in individuals, the mean serum levels of these laboratory markers did not significantly change before treatment initiation and in the final follow‐up (p‐value >0.05). The percentage changes in the mean serum levels of laboratory markers, BMI, weight, and waist circumference were very similar between patients with metabolic syndrome and other patients, and there was no significant difference between them (p‐value >0.05). However, in a study by Parinitha et al., abnormal liver enzymes were observed in 6% of patients, and abnormal serum lipid levels were observed in 6% of patients 3 months after receiving isotretinoin.^20^ There are studies that demonstrate that the use of isotretinoin can disrupt the levels of lipoproteins and serum lipids in individuals.^24^, ^25^ On the other hand, there are studies that show the negative effects of isotretinoin on laboratory markers were so minimal that they were completely negligible and did not require monitoring.^26^, ^27^ This difference in findings may be due to differences in the prescribed dosage of the drug. The use of isotretinoin with a lower dosage provides a good balance between effectiveness and the associated side effects.^28^, ^29^

In a study conducted by F Çetinözman et al. in 2014 in Turkey, titled “Insulin Sensitivity, Androgens, and Isotretinoin Treatment in Women with Severe Acne,” the levels of insulin and androgen sensitivity before and after treatment with isotretinoin were examined. The variables in this study included androgen levels, lipids, glucose, and insulin levels, which were evaluated in 26 patients with severe acne and 21 healthy individuals during insulin resistance testing. Among these individuals, 20 patients received isotretinoin treatment for a minimum of 6 months. The results of this study showed that all the variables examined in both the control and experimental groups were initially similar in magnitude. After receiving isotretinoin treatment, it was demonstrated that this medication increased body mass index and triglyceride levels without affecting insulin or androgen sensitivity.^30^ However, in our study, there were no significant changes in the mean laboratory factors, including LDL, HDL, triglycerides, and BSBS, between individuals before treatment and at the final follow‐up (p‐value >0.05).

In a study conducted by DT Ertugrul et al. in 2011 in Turkey, titled “The Use of Isotretinoin Does Not Induce Insulin Resistance in Patients with Acne,” a clinical trial was performed involving 48 patients with acne vulgaris who were evaluated before and after 3 months of isotretinoin treatment. The variables to be examined were measured before the trial initiation and 4 months after receiving isotretinoin treatment. The measured parameters included insulin, C‐peptide, FBS (fasting BS), ALT and AST (liver enzymes), total cholesterol, triglycerides, HDL, LDL, and VLDL. The results of this study showed a significant increase in AST, ALT, total cholesterol, LDL‐C, and triglycerides after the use of isotretinoin, while there was no significant increase in FBS, insulin, and C‐peptide levels.^31^ However, in our study, there were no significant changes in the mean laboratory factors, including LDL, HDL, triglycerides, and BS, between individuals before treatment and at the final follow‐up.

In a study conducted by BC Cemil et al. in 2016 in Turkey, 32 patients were enrolled. Oral isotretinoin was initiated at a dose of 0.5–0.6 mg per kg of body weight and increased to 0.6 to 0.75 mg per kg. The variables measured in this study were evaluated before the treatment intervention and 3 months after the start of treatment. The results of this study showed that there was no significant change in body mass index before and after the treatment.^32^ These results were consistent with the results of our study. In our study, there was no significant difference in the BMI of individuals between the initial assessment and the final follow‐up period.

In a study conducted by Rasi et al. in 2014, with the aim of investigating the effect of low‐dose daily isotretinoin in patients with moderate to severe acne, 146 patients with acne prone to scarring were treated with a regimen consisting of 20 mg of isotretinoin per day, up to a total cumulative dose of 120 mg per kilogram. At the end of the treatment period, 96.4% of the patients demonstrated complete clearance of their acne. During the 5‐year follow‐up, relapse occurred in 11 patients (7.9%). All side effects were mild, and discontinuation of treatment was not necessary, indicating that the use of low‐dose isotretinoin can be a safe and well‐tolerated treatment option without significant adverse effects (including liver‐related complications).^27^ In our study, it was the same. The results of our study indicate that the use of oral isotretinoin as a therapeutic option for managing acne vulgaris can be a safe and low‐adverse‐effect choice, provided that the drug dosage is properly adjusted.

## 5 CONCLUSION

Isotretinoin, as an effective treatment option for acne in standard doses, does not have metabolic side effects such as weight changes, BMI, and other factors related to metabolic diseases like BS levels, triglyceride levels, LDL levels, HDL levels, and so on.

### 5.1 Limitations

Among the limitations of our study are the small number of participants, the follow‐up intervals of patients, and the evaluation of parameters at 1 month and 3 months; however, the long‐term assessment of isotretinoin consumption was not included. Therefore, it is recommended that future studies be conducted with a larger sample size and longer follow‐up periods.

## AUTHOR CONTRIBUTIONS

A.S and F.D designed the study. A.S, A.J and F.D wrote the paper. S.S AND A.J edited the manuscript. All authors have read and approved the content of the manuscript.

## CONFLICT OF INTEREST STATEMENT

The authors have no conflict of interest to declare.

## ETHICAL APPROVAL

The researchers were committed and adhered to the principles of the Helsinki Convention and the Ethics Committee of the Iran University of Medical Sciences in all stages.

## CONSENT STATEMENT

After providing the necessary explanations, written informed consent was obtained from the patient regarding the submission of their clinical condition to medical journals. Additionally, the patient has been assured that their name and personal details will be kept confidential by the authors.

## TRANSPARENCY DECLARATION

Authors declare that the manuscript is an honest, accurate, and transparent. No important aspect of the study is omitted.

## Data Availability

All data produced in the present study are available upon reasonable request to the authors.
